# Mechanisms driving spatial distribution of residents in colony biofilms: an interdisciplinary perspective

**DOI:** 10.1098/rsob.220194

**Published:** 2022-12-14

**Authors:** Lukas Eigentler, Fordyce A. Davidson, Nicola R. Stanley-Wall

**Affiliations:** ^1^ Division of Molecular Microbiology, School of Life Sciences, University of Dundee, Dundee DD1 5EH, UK; ^2^ Mathematics, School of Science and Engineering, University of Dundee, Dundee DD1 4HN, UK

**Keywords:** spatial distribution, colony biofilms, genetic drift, founder density, microbial interactions

## Abstract

Biofilms are consortia of microorganisms that form collectives through the excretion of extracellular matrix compounds. The importance of biofilms in biological, industrial and medical settings has long been recognized due to their emergent properties and impact on surrounding environments. In laboratory situations, one commonly used approach to study biofilm formation mechanisms is the colony biofilm assay, in which cell communities grow on solid–gas interfaces on agar plates after the deposition of a population of founder cells. The residents of a colony biofilm can self-organize to form intricate spatial distributions. The assay is ideally suited to coupling with mathematical modelling due to the ability to extract a wide range of metrics. In this review, we highlight how interdisciplinary approaches have provided deep insights into mechanisms causing the emergence of these spatial distributions from well-mixed inocula.

## Graphical abstract

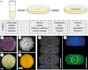

## 1. Introduction

Microbes possess the propensity to form dense communities, called biofilms, in which the individual cells become embedded in self-produced extracellular matrix molecules [1]. The extracellular matrix typically consists of extracellular polysaccharides, extracellular DNA, proteins, lipids and components of lysed cells [1–5]. The residents of biofilms are remarkably robust to environmental stresses, for example displaying increased antimicrobial tolerance, and thus present microbes with a selective advantage compared to planktonic counterparts [5–8]. Biofilms can significantly affect their surrounding environment and thus play substantial roles in many environmental, industrial and medical settings. For example, biofilms are fundamental to the correct functioning of the human gastrointestinal tract [9], are used in wastewater treatment [10], are implemented as biofertilizers and biopesticides in agriculture [11,12] and are essential to global biogeochemical cycling [13,14]. However, biofilms also present noteworthy challenges to human life and are a known cause of antibiotic-resistant and tolerant infections [15], and to be responsible for the fouling of medical [16] and industrial devices [17].

The ubiquitous occurrence and impact of biofilms have led to broad ranging efforts to advance our understanding of this key form of microbial life. Biofilm structure can be affected by the properties of biofilm constituents that are made in response to the experienced environmental conditions [18,19]. As a consequence, many *in vitro* methods have been established to explore how biofilm structure emerges under a given set of environmental pressures in laboratory conditions [3]. Some approaches aim to replicate the natural environment of specific biofilms, such as in wound-healing [20], wastewater treatment plants [21], or plant-root colonization within the rhizosphere [22]. Other common approaches take a reductionist approach to allow the ready intersection of genetics with biofilm analyses and the elucidation of general principles. These methods include the investigation of submerged biofilms under conditions of flow using the ‘flow cell biofilm system’ [23], the study of submerged biofilms that attach to abiotic surfaces under static conditions using the microtiter dish biofilm assay’ [24,25], the growth of floating biofilms called ‘pellicles’ on the liquid–air interface [3] and the ‘colony biofilm assay’ where cells are grown on a solid–air interface on agar plates [3].

**Figure 1.**
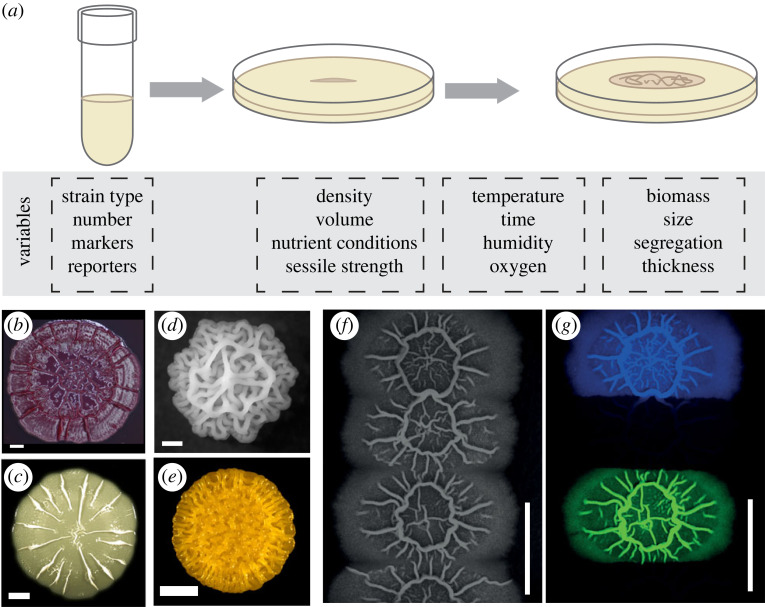
The colony biofilm assay. (*a*) Schematic of the colony biofilm assay, controllable variables and observable outcomes of the experimental assay. (*b–e*) Colony biofilms of different species feature a wide array of appearances. *P. aeruginosa* (*b*), *E. coli* (*c*), *S. cerevisiae* (*d*), *V. cholerae* (*e*) all form colony biofilms with visible striking, architecturally complex macroscale structures. (*f–g*) Strains producing fluorescent proteins can reveal hidden spatial distributions of residents. A *B. subtilis* colony biofilm, spotted using four separated inocula with residents producing mKate (blue), no fluorescent protein, GFP (green), and no fluorescent protein (from top to bottom) is shown under brightfield light in (*f*). In (*g*), only fluorescent signals of the same biofilms are visualized. The strains used were derivatives of *B. subtilis* isolate NCIB 3610. The colony biofilms were grown on MSgg agar (1.5% w/v) for 48 h incubation at 30°C prior to imaging (see [26] for a full description of the method). The scale bars are 1 mm, 5 mm, 1 mm, 2 mm, 10 mm and 10 mm in (*b*–*g*) respectively. Sources: (*b*) is from [27]; (*c*) is from [28] in accordance with its Creative Commons Attribution License; (*d*) is from [29] in accordance with its Creative Commons Attribution License; (*e*) is courtesy of Anna Potapova and Fitnat H. Yildiz; (*f*–*g*) are unpublished images from the Stanley-Wall laboratory.

The colony biofilm assay is a powerful and widely used method of investigation. In this assay, founding cells are deposited on an agar-solidified medium that supplies the microbes with nutrients (figure 1*a*). The cells are incubated in controlled conditions that allow growth of the inoculum and eventual development of an architecturally complex macroscale structure (figure 1*a–e*). Examples of applications of the colony biofilm assay are diverse, and include studies to examine interactions between multiple biofilms [30–34], reveal emergent properties of dual-strain biofilms that cannot be predicted by monocultures [35], elucidate regulatory pathways responsible for biofilm formation [36,37] and identify biofilm matrix constituents [38] to name but a few. Investigations using the colony biofilm assay are also ideally suited to interrogation by mathematical models (box 1). This is due to the rich suite of quantifiable assay outcomes, such as strain abundance [26], degree of spatial segregation [38,80], biofilm footprint [81], degree of spatial segregation [38,80], biofilm footprint [81], growth rate [82,83], thickness [82,83], viscoelastic properties [57,59], competitive fitness [57,59], mutation rates [57], and biomass [44,84], which are connected to experimentally controllable parameters (figure 1*a*).

Box 1. **Mathematical models reveal hidden spatial structure in colony biofilms.**

Applications of the colony biofilm model often benefit from hypotheses-generation via mathematical modelling [39,40]. Theoretical frameworks significantly simplify biofilm growth dynamics. However, careful formulation can transform these frameworks into powerful predictive tools. Mathematical models can simulate biofilm growth in seconds and are easily customizable to an extent that cannot be achieved with experimental systems. In broad terms, three fundamentally different model types, each with its unique advantages and disadvantages, have been applied in this context: continuum models, normally comprising partial differential equations (PDEs); individual-based models (IBMs); and cellular automata (CA) (table 1).

PDE models represent microbes (and other model constituents of interest) as densities (e.g. weight per unit volume) that are distributed in a continuous spatio-temporal environment. Growth dynamics are represented typically by deterministic (i.e. non-random) functional responses that are derived from averages across many individual cells. Therefore, PDE models are ideally suited to study large-scale population dynamics. Deterministic PDEs can also be extended to capture large-scale random effects through additional noise terms (stochastic PDEs – SPDEs) [54]. However, PDEs are incapable of investigating finer-scale phenomena that result from random differences between individual cells, such as genetic drift [40,52]. PDE models are computationally inexpensive (compared with IBMs) and tools from mathematical analysis facilitate analytical tractability that can provide deep insights into the underlying processes without the need for large numbers of model simulations across a parameter space [50,52,70]. Numerical investigations of PDE models typically rely on discretization of space and time via finite difference or finite-element methods. Software packages, such as Matlab's PDE Toolbox [71], C++ based FreeFEM [72] and Python-based FEniCS [73,74], facilitate implementation.

IBMs represent bacterial cells as idealized individual agents whose dynamics are governed by a set of rules describing cell responses to their environment. These rules are iteratively applied to update the system state. Environmental factors, such as biofilm matrix elements and nutrients, can also be described by individual agents, but are sometimes resolved by PDEs [58]. IBMs can resolve agents and their dynamics in continuous space. This requires considerable computational power, but enables tracking of specific spatial cell properties, such as cell shape or orientation [49]. A particularly powerful advantage of IBMs is their ability to consider microscopic details [49] and stochasticity underpinning single-cell dynamics [46,57,65]. For example, cell division rates can be chosen individually for each cell from a data-informed probability distribution [42]. However, due to computational restrictions, the total number of agents that can be used in an IBM is currently restricted. Therefore, agents typically either represent single cells when, for example early stages of microbial aggregation are being investigated, or clusters of cells when larger structures are being studied. IBMs can be implemented in any commonly used programming language, but software such as CellModeller [75], Nanoverse [42,76], NUFEB [77] and iDynoMiCs [78], enable manipulation of existing frameworks and implement computationally efficient algorithms.

CA share many features with IBMs, but with one important difference: spatial dynamics are resolved on a lattice. Each lattice point is fixed in space and is assigned a state from a discrete set of possible states (e.g. empty, occupied by species 1, occupied by multiple species). Unlike in IBMs, lattice points do not represent single cells, but rather correspond to small area elements of a biofilm. Therefore, the set of possible states can include settings in which more than one species is present at a lattice point. Lattice points connect, allowing for interactions between lattice points. This discretized representation of space results in considerably less computational cost of implementations compared with IBMs. However, specification of a lattice creates anisotropy in the spatial domain and for example, causes lengthening of the *in silico* biofilm along the axes of the lattice [79]. Therefore, the use of CA to study radial biofilm growth has to be treated with caution. Thanks to major recent advances in the availability of computational power, IBMs are often more suitable.

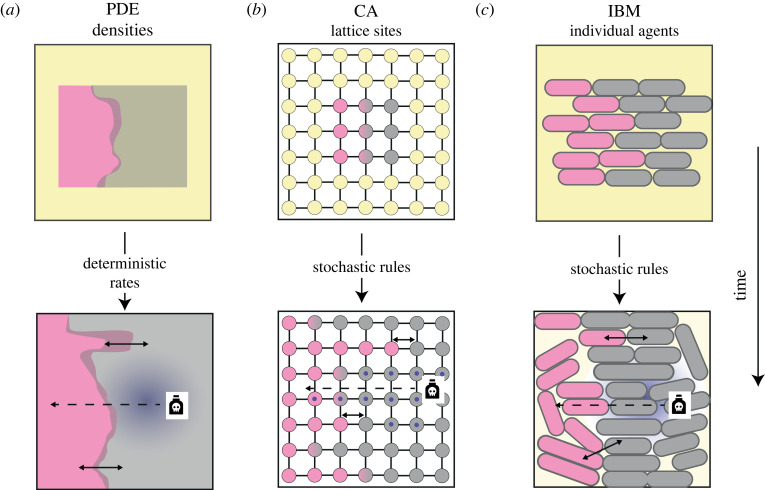

**Figure BOX**. Mathematical models used to reveal hidden spatial structures in colony biofilms. Three types of model are commonly used to reveal hidden spatial structures and interactions between residents in colony biofilms. In these schematics, two strains are visualized in pink and grey colour, nutrients are depicted in yellow, and a diffusible toxin is shown in blue (either as a ‘cloud’ in (*a*) and (*c*) or as a ‘dot’ in (*b*)). Contact-dependent (short-range) interaction mechanisms are highlighted through solid lines; contact-independent (longer range) mechanisms are visualized by dashed lines. In CA, interaction between strains can occur at a single lattice point (if in a state representing occupation by different strains), between lattice points if points are connected (black lines) or indirectly via diffusible molecules (here blue dots) that can act across multiple lattice points.

(*a*) PDEs. Cells are represented as densities (e.g. number of cells per unit area) for each resident. Overlap between populations is possible (dark pink). The state of each density is updated in continuous time through deterministic rules with prescribed rates. (*b*) CA. Space is discretised into a lattice representing area elements in which each lattice point is assigned a state (e.g. nutrient only, occupied by one strain, occupied by two strains, and toxin present/absent). Lattice points can, but do not necessarily, represent single cells. States are updated over discrete time-steps through stochastic rules like those used in IBMs. (*c*) IBMs. Each cell is represented as an individual agent in continuous space and assigned to one type of biofilm resident. Over discrete time-steps, the state of each cell is updated through stochastic rules and new cells can emerge through cell division processes. Each individual can be associated with a shape and orientation that can change over time.

Colony biofilms are visually striking objects; however, the study of spatial structure in colony biofilms is not limited to their *visible* topological features. The use of genetically modified strains that produce a fluorescent protein (e.g. GFP etc.) can reveal *hidden* spatial distributions of residents (figure 1*f–g*). These patterns can include dynamic transcriptional profiles across the landscape of the colony biofilm, resulting from the division of labour or heterogeneous responses to local conditions [85]. The patterns can also include the spatial distribution of separate cell lineages originating from the founding cells in the inoculum. Various measures to quantify both visible and hidden spatial structures exist, including but not limited to Ripley's K function to quantify heterogeneities during initial colony growth [86], spatial assortment [43], neighbour [87] and intermixing indices [66] to determine the degree of segregation among strains, Shannon's diversity index to quantify species diversity [45,65], and fractal dimension to measure the extent of dendritic patterns [87]. Many of these measures are implemented in biofilm image analysis software such as BiofilmQ [88] and Comstat [89,90] and are therefore readily available for application in future research.

The spatial distribution of residents in colony biofilms has a significant impact on interaction mechanisms between the cells over long *evolutionary* timescales spanning multiple iterations of the biofilm life cycle [91–93]. For example, cooperative traits within a genotype, such as the production of public and common goods, are selected for by spatial segregation between genotypes [43,56,94–96], while spatial mixing leads to the evolution of competitive genotypes [45]. The impact of spatial distributions on the evolution of interaction mechanisms in biofilms has previously been discussed [97]. The focus of this review is on events that occur over the shorter timescale (sometimes referred to as *ecological* timescale even though evolutionary dynamics may also be involved) of the growth of a single biofilm, in which cell-to-cell interactions play a crucial role in the transformation of an initially well-mixed and randomly placed inoculum into colony biofilms displaying intricate spatial distributions. Many of the mechanisms uncovered have used interdisciplinary approaches comprising mathematical modelling and microscopy to monitor, quantify and hence better understand the spatial expansion of separate cell lineages from the cells in the inoculum (box 1).

## 2. The different scales of interactions in colony biofilms

**Figure 2.**
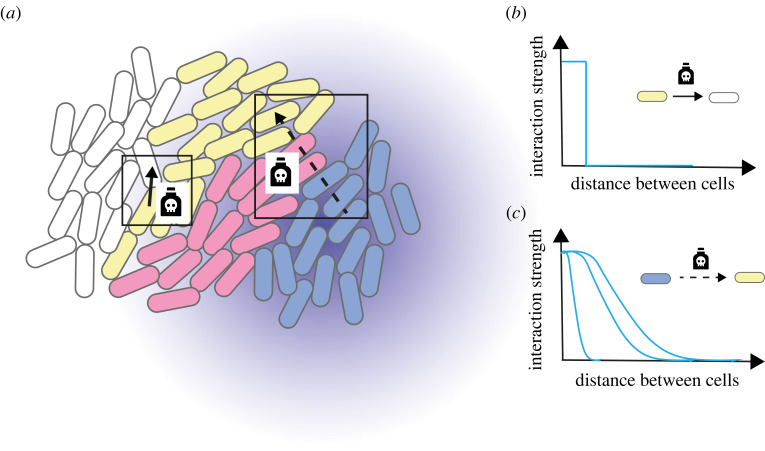
Interaction scales in colony biofilms. (*a*) Schematic of different interaction scales in colony biofilms comprising different residents. Contact-dependent interaction mechanisms only impact populations if associated residents are in direct contact (e.g. yellow can kill white but not blue even if blue is susceptible to contact-dependent killing by yellow). Contact-independent mechanisms act even if residents are not in direct contact (e.g. blue can kill yellow if yellow is susceptible to contact-independent killing by blue). (*b,c*) Interaction strengths depend on distance between cells and type of interaction mechanism. Contact-dependent mechanisms act only if cells are sufficiently close (*b*), while the interaction strength of contact-independent mechanisms decreases with increasing distance between cells (*c*).

Interactions that occur between residents of a colony biofilm fundamentally depend on the scale of interaction mechanism(s) and the scale of the spatial mixing (figure 2*a*) [98]. The term *spatial mixing* typically refers to random mixing of residents. However, the term also describes spatial patterns at the microscale, such as dendritic patterns or fractal-like structures [66–68].

In turn, cell division, spatial expansion and the dynamics of cell-to-cell interactions all contribute to cell lineages in colony biofilm either being spatially mixed or arranged in an intricate segregation pattern at the macroscale. For example, a specific cell lineage can affect neighbouring, genetically different cell lineages through contact-dependent mechanisms (e.g. localized sharing of public and common goods (figure 2*b*) [99] or by the type VI secretion system [100,101]), but can also interact with cells beyond their immediate vicinity through the release of quorum-sensing signalling molecules [102] or the secretion of diffusible toxins [103], (figure 2*c*). Provided interaction mechanisms act on a shorter scale than that of distribution patterns, spatial segregation offers protection from antagonistic interactions [57]. Spatial segregation within distribution patterns is dynamic and can be generated rapidly from the formation of thin boundary layers (with a thickness on the scale of single cells) of dead or growth-inhibited cells that physically protect other targets [57]. The colony biofilm model is used to reveal mechanisms enabling organization of biofilm residents into these intricate distribution patterns. Such mechanisms can be actively regulated by the interacting populations or can be induced by exogenous factors.

## 3. The importance of the biofilm inoculum to distribution patterns

**Figure 3.**
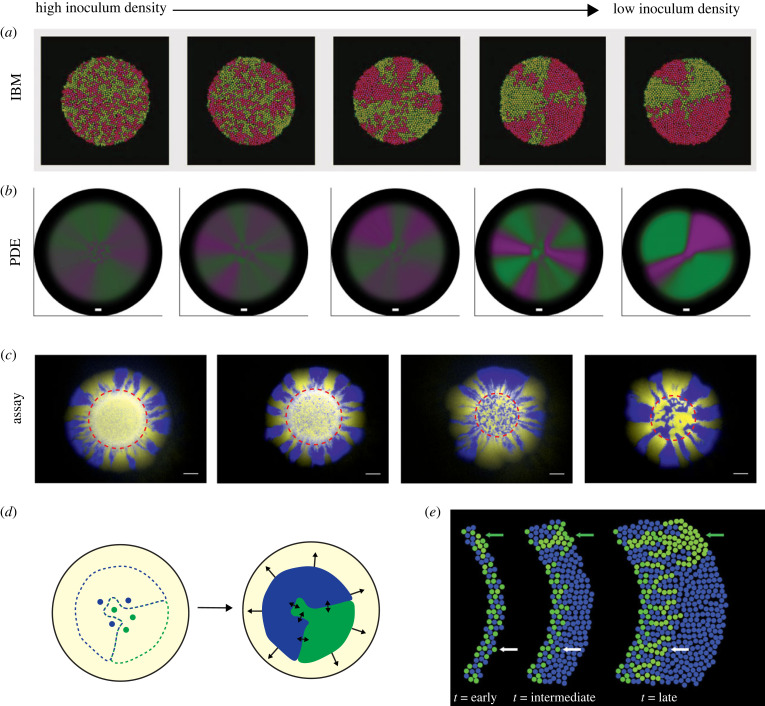
Low inoculum densities induce spatial segregation. (*a–c*) Simulations using IBMs (*a*) and PDEs (*b*), and realizations of the colony biofilm assay using two isogenic strains of *E. coli* (*c*) all show an increase in spatial segregation between residents with decreases in the density of cells in the biofilm inoculum (decreasing from left to right). Note that other mechanisms (such as spatial genetic drift) can induce segregation in the periphery of high inoculum density biofilms ((*c*), outside red dashed circle). The scale bar in (*c*) is 1 mm long. (*d*) Schematic of the contribution of initial CFUs. Only CFUs that have access to free space (i.e. are not surrounded by other CFUs) can make significant contributions to biofilm expansion. Once established, little movement of internal sector boundaries occurs (indicated by length of black arrows), even if residents interact antagonistically. (*e*) The relative distribution of residents in the inoculum determines the orientation of dendritic patterns in mature biofilms. Sources: (*a*) is adapted from [43]; (*b*) was obtained using numerical code published in [26]; (*c*) is adapted from [57] in accordance with its Creative Commons Attribution (CC BY 4.0) license; (*e*) is from [68] in accordance with its Creative Commons Attribution License (CC BY 4.0).

Investigations of interactions between biofilm residents typically make use of the colony biofilm model by using a high-density inoculum in which the initial relative abundance of each resident can be controlled. Depending on the volume and density of the inoculant, the number of colony-forming units (CFUs) in the inoculum can reach the order of millions [26]. However, the inoculum density can significantly affect the spatial distribution of biofilm residents and thus the interactions among them in mature colony biofilms. This topic has been explored through experimental assays and three commonly used types of mathematical models: individual-based models, cellular automata and systems of partial differential equations (box 1). Reductions in inoculum density lead to increased spatial segregation (i.e. a reduction of exposure of one resident to others) in the corresponding mature biofilms across a variety of species, independent of the interaction mechanism (figure 3*c*) [26,41–43,57,58,60]. Consequently, if interaction dynamics are underpinned by contact dependent or locally acting antagonisms, then low inoculum densities provide a lifeline for a weak competitor to persist in the community [41,42,57]. By contrast, spatial separation induced by low inoculum densities prevents exploitation of cooperative interactions by ‘cheating’ residents [43]. It has been hypothesized that the increase in spatial separation in mature biofilms occurs because, on average, the size that a clonal aggregate (originating from a single CFU) reaches before it interacts with those of other residents, increases with decreasing inoculum density [41–43]. However, no empirical or theoretical validation of this hypothesis is available to date. By contrast, tracking of cell lineages in a species-independent mathematical model has revealed that a focal CFU gains advantage from large distances to competitor CFUs only if it is free to expand radially during biofilm growth (figure 3*d*) [26]. This is because, in the absence of antagonistic interactions, cell lineages of different residents do not merge (under certain conditions) [104] and expansion of a resident's footprint during later stages of biofilm growth is therefore restricted to cell division within the expansion front (figure 3*d*) [53]. Therefore, microbes initially located within the edge of the biofilm inoculum contribute significantly more to radial growth than those found in the centre of the inoculum. This also highlights the impact of the ‘coffee ring effect’ which describes physical forces that lead to accumulation of cells within the edge of the inoculum as the inoculum dries [105,106]. To this date, the precise impact of this effect on spatial distribution of cell lineages in biofilms remains understudied.

The importance of the biofilm inoculum is not exclusively restricted to those inoculated with low cell numbers. For example, in an experiment that considered a community of two metabolically interdependent *Pseudomonas stutzeri* strains inoculated at high density (and in a corresponding individual-based model), the relative positions of single cells within the inoculum edge determined the orientation of the resulting dendritic patterns in the mature biofilm, which in turn led to heterogeneities in the speed of radial expansion within a single-colony biofilm [68] (figure 3*e*). Similarly, in a system of three interacting strains of *Escherichia coli,* early community composition was found to be an excellent predictor of long-term population dynamics [107]. Both these examples represent cases in which the mature community can be mapped accurately to its inoculum. This equips the associated mathematical models with significant predictive power [26,68]. In theoretical frameworks, the inoculum can easily be manipulated (e.g. the cells can be arranged into a predefined pattern) which facilitates rapid hypothesis generation. However, laboratory-deployed ‘cell printing’ methods, in which cells can be deposited in a controlled distribution pattern, are actively being researched and may in the future enable experimental inoculum manipulation akin to that currently only afforded by mathematical modelling [108–112].

## 4. Spatial genetic drift induces spatial segregation

**Figure 4.**
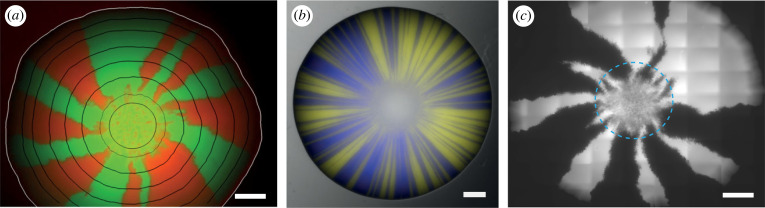
Spatial genetic drift induces spatial segregation in colony biofilms. (*a*) Two isogenic *E. coli* strains spatially segregate into a small number of sectors with jagged sector boundaries. (*b*) Two isogenic *S. cerevisiae* strains spatially segregate into many sectors with smooth sector boundaries. (*c*) Two isogenic *E. coli* strains spatially segregate into a small number of sectors with jagged sector boundaries. All scale bars are 1 mm long. Note the difference in direction of chirality between (*a*) and (*c*), which could be due to differences in the point of view (from top versus from bottom). Sources: (*a*) is adapted from [113] (Copyright 2007 National Academy of Sciences); (*b*) is from [54] (Copyright 2014 National Academy of Sciences); (*c*) is adapted from [114] in accordance with its Creative Commons Attribution-NonCommercial-NoDerivs 4.0 International License.

Spontaneous segregation of originally well-mixed cell lineages in colony biofilms can also emerge from high-density inoculants due to *spatial genetic drift* of the residents [45,46,113]. Spatial genetic drift refers to variations of resident diversity along the biofilm edge caused by temporary differences between single cells that are induced by randomness in the cell division process [46,48,113] (figure 4). Provided nutrients are low in concentration and unable to diffuse towards the biofilm centre, only cells within a thin band located at the biofilm edge actively contribute to radial biofilm growth [53,55,67]. For example, when *E. coli* is grown on LB plates (solidified with 1.5% (w/v) agar), the width of this active band at the periphery has been estimated to be 30 µm [113] and even narrower regions (15 µm) of active growth have been reported from *Pseudomonas aeruginosa* biofilms exposed to poor-nutrient conditions [53]. Therefore, temporary advantages of single cells within the active band can lead to the onset of monoclonal sectors during early stages of biofilm development [45,48,49,55,113]. These temporary advantages are caused by randomness in cell growth and division, a well-known bet-hedging strategy of microbes [115,116]. These random temporary advantages even allow fixation of monoclonal cell lineages without a selective (fitness) advantage [113]. Indeed, the number of mutations that persist in biofilms is higher than in well-mixed communities [47]. During later growth stages, coarsening dynamics lead to a gradual reduction of the number of sectors within the biofilm edge [44,48]. Collectively, this leads to a characteristic distribution pattern in which residents remain mixed within the footprint of the inoculum of the biofilm, but spatially segregate elsewhere [51,113]. The mixing of residents within the biofilm centre distinguishes this distribution pattern from that emerging from low-density inocula (figures 3*a–c* and 4).

Genetic drift has been shown to facilitate segregation in colony biofilms between residents in a wide range of organisms, including *E. coli* [49,51,113,114], *Saccharomyces cerevisiae* [49,54,56,113], *Bacillus subtilis* [117], *Schizosaccharomyces pombe* [49], *P. aeruginosa* [51,53,118], *Pseudomonas protegens* [118], *P. stutzeri* [66,67] and *Klebsiella pneumoniae* [118], with differences in the shape of sector boundaries, which are determined by species composition, extracellular matrix elements and environmental conditions [46,50,51,114,118]. For example, in *K. pneumoniae* and *S. cerevisiae,* sector boundaries between cell lineages are smooth and, in many cases, straight lines [54,113,118], while in *P. protegens, P. stutzeri* and *E. coli,* (and computationally modelled communities of rod-shaped cells) boundaries have a jagged appearance [41,113,118] (figure 4*a–c*). Computational modelling alongside experimental assays involving *E. coli* mutants with different cell shapes have suggested buckling of initially aligned rod-shape cells as a likely cause of jagged boundaries for rod-shaped strains [119]. However, the universality of this theory is questioned by observations of straight boundaries in biofilms of other rod-shaped strains, which is attributed to secreted extracellular matrix molecules [118]. Moreover, the absence of extracellular constituents increasing cell-to-cell and cell-to-substratum adhesion, and cell filamentation cause significant chirality in the sector boundaries [114,120].

Mathematical modelling has yielded additional insights into the underpinning segregation dynamics through the manipulation of system properties that is difficult or impossible to achieve in experimental settings [45,49,51,53–56,67]. For example, theoretical frameworks have shown that lack of segregation occurs in the absence of nutrient limitation [53] and an increase in segregation is predicted with a decrease in nutrient diffusivity [55].

Combined, spatial genetic drift is regarded as a universal promoter of genotypic diversity in laboratory-grown colony biofilms. However, genetic drift-induced segregation can be suppressed by strong mutualisms between residents [54,67] or spatial environmental heterogeneities that e.g. slow the growth of individual cells with a reproductive fitness advantage [48]. Therefore, it is currently not clear how relevant this mechanism is to diversity in biofilms growing in natural, and hence heterogeneous environments.

## 5. Colonization abilities compensate for lack of local competitiveness

The spatial component of interaction mechanisms in colony biofilms has the potential to create ecological niches that prevent one resident from taking over the community despite an innate fitness advantage under planktonic conditions. A classical tenet of theoretical ecology asserts that trade-offs between local competitiveness and colonization abilities stabilize coexistence [121–124]. Recent applications of the colony biofilm model verified that this principle indeed applies to biofilms. Residents with a superior ability to expand radially are capable of encircling (potentially locally superior) competitors during initial growth stages, thus blocking further expansion of competitors [58,80,118]. Mechanisms that enable a cell lineage to gain preferential access to the biofilm edge include faster growth rates [57], flagella-based motility [118], production of mucoid matrix elements [59] and exploitation of extracellular matrix components produced by competitors (cheating) [80]. Moreover, a recent study of colony biofilms of two strains of *Raoultella planticola* highlighted the potential occurrence of complex synergies between biofilm expansion rates and competitive interactions [60]; a strain that expanded at a slower rate in monoculture exhibited a local increase in expansion speed when interacting with a competitor strain and thus gained preferential edge access over time [60]. Moreover, even if genetically different cell lineages interact antagonistically, radial expansion of sectors dominates over invasion processes along internal sector boundaries [26], due to the microbes' limited ability to penetrate existing competitor sectors [52,104] (figure 3*d*). The randomness underpinning cell positions in the inoculum leads to, on average, more uneven distributions along the boundary of the region encircling the initial CFUs for low cell numbers and thus an increase in spatial segregation. These theoretical predictions that the potential for radial expansion is the determinant of spatial segregation, rather than initial surface colonization within the inoculum, are supported by empirical work using a *P. aeruginosa* model system in which the impact of the inoculum density only becomes apparent as the biofilm matures [58].

Cell shape also has a significant impact on residents gaining and retaining access to the biofilm edge, with elongated cells possessing a clear advantage [58,61]. These studies represent another prime example of the advantages afforded by mathematical modelling. In one of the reported studies, it was impossible to experimentally manipulate cell shape without affecting growth rate or other properties [61]. However, mathematical modelling has been capable of investigating the impact of cell shape in isolation on expansion dynamics. This approach predicted a clear advantage of rod-shaped cells due to their orthogonal alignment to the biofilm edge and consequent exclusion of coccoid-shaped cells from the edge [58]. Additional analysis of a mathematical model in which a large range of parameter values was tested, revealed that even slight expansion advantages enable a resident with inferior antagonistic capabilities (local competitiveness) to persist in the population [26]. This was due to the large impact on competitive fitness of small differences in colonization abilities [57].

In summary, the results concerning the importance of spatial expansion over antagonistic actions confirm theoretical evolutionary studies of range expansion (the process of a species’ expansion into previously unoccupied territory), which revealed that dispersal traits are selected for during range expansion over traits guaranteeing competitive success under well-mixed conditions [125,126].

## 6. Syntrophy induces microscale patterns

General evolutionary theory suggests that cooperative interactions are favoured by mixing among biofilm residents [97]. Applications of the colony biofilm model have recently revealed that symbiotic metabolic relationships between cell lineages impose spatial mixing through signature dendritic microscale distribution patterns in biofilms [44,66–68]. Syntrophy, sometimes referred to as cross-feeding, describes metabolic dependency between cell lineages and is common among microbes [127] and in particular within biofilms [4,128]. In syntrophic relationships, a ‘producer’ strain reduces a nutritional resource into a metabolite that facilitates the growth of a ‘consumer’ strain unable to process the originally supplied resource [129,130]. Such commensal interactions are sometimes referred to as weak mutualisms to distinguish from strong mutualisms in which the produced metabolite is toxic to the producer. In the latter case, syntrophy leads to detoxification of the producer's environment [44,63,67]. Moreover, syntrophic relationships are not necessarily unilateral; strains can be ‘producer’ and ‘consumer’ (of different metabolites) simultaneously [54].

Biofilm expansion of strains in a unilateral syntrophic relationship is successive; the producer leads expansion along the biofilm edge because the consumer can only grow in locations at which the producer has released sufficient amounts of metabolites [66]. Such temporal heterogeneity in nutrient availability is reminiscent of cell-autonomous nitrogen oscillations in single-strain *B. subtilis* colony biofilms that lead to the arrangement of spores in a ring-like distribution pattern [62]. A different spatial distribution pattern emerges for a pair of metabolically interdependent *P. stutzeri* strains. The consumer strain forms dendrites due to mechanical forces exerted by producers, which leads to an increase in mixing between strains compared to the footprint of the inoculum [66]. Moreover, while the detoxifying impact of strong mutualistic syntrophies is beneficial to the biofilm community [63,64], even weak commensal syntrophies can lead to increases in biomass production compared to single-strain biofilms, because metabolic products are more efficiently used [84]. This outcome is in agreement with results on other cooperative behaviours that were highlighted to be beneficial to the productivity of genetically diverse biofilm communities [118]. Generally, cooperative behaviours are prone to exploitation by non-cooperating cheaters [99]. However, both theoretical and experimental implementations of the colony biofilm assay have shown that the spatial distribution patterns within colony biofilms offer protection to syntrophic dynamics due to spatial exclusion of cheaters from sites of high nutrient availability [65,69].

**Figure 5.**
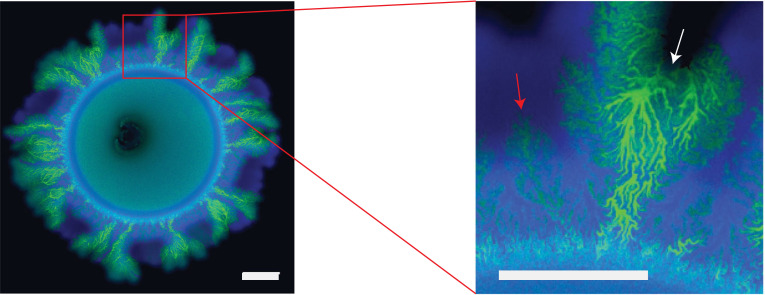
Syntrophic interactions lead to dendritic patterns and pattern diversification. A biofilm comprising two strains of *P. stutzeri* engaged in a syntrophic relationship is shown. The blue resident (producer) reduces the externally supplied nitrate ${(\;\mathrm{NO}}_{3}^{-})$ to nitrite ${(\;\mathrm{NO}}_{2}^{-})$. The green resident (consumer) is unable to metabolize nitrate but capable of using the produced nitrite. This metabolic interdependency results in two distinct dendric patterns: ‘producer-first’ pattern, in which the producer leads the radial expansion and the consumer forms outwards-facing dendrites behind the expansion front (red arrow), and ‘consumer-first’ patterns in which the consumer is pushed along the biofilm edge by the producer and forms inwards-facing dendrites through subsequent inward growth (white arrow). The scale bar is 1 mm long. Source: adapted from [68] in accordance with its Creative Commons Attribution (CC BY 4.0) license.

Successive expansion induced by unilateral syntrophy also leads to diversification of distribution patterns, i.e. the emergence of two distinct microscale distribution patterns within the same biofilm: dominance of producers along the biofilm edge or the rare appearance of consumer dendrites within the biofilm edge that eventually leads to gradual emergence of monoclonal consumer sectors along the periphery [44,63,68] (figure 5). An understanding of this phenomenon is provided by mathematical models with the ability to track a range of system quantities, such as historical growth rates and nutrient distributions, and an arbitrarily large number of cell lineages [44,63]. This analysis revealed that due to an initial lack of the produced metabolite, most consumer cells lose access to the biofilm edge. However, a small number of single-consumer cells are *passively* pushed towards the edge by producer cells [63,68]. During later growth stages, consumer cells benefit from a growth advantage due to metabolite availability, and descendants of the single-consumer cells within the boundary can form large monoclonal sectors, provided metabolite diffusion is sufficiently high [44,68]. Toxicity of the produced metabolite to the producer can cause particularly large differences in growth rates during later stages of biofilm growth and thus lead to the establishment of a larger number of monoclonal consumer clusters [63]. In summary, even cooperative syntrophic interactions lead to spatial segregation during later stages of colony biofilm growth, following the occurrence of fractal-like intermixing during early growth (figure 5).

## 7. Outlook

The colony biofilm model has proved to be critical to disentangling the complex hidden structures and interaction dynamics among residents in biofilms. Nevertheless, the model currently features several limitations that require to be resolved to further extend our knowledge of resident distribution patterns in colony biofilms. Many investigations using the colony biofilm assay assume that the biofilm is essentially a flat object. From one perspective this is a reasonable assumption as the biofilm's thickness is orders of magnitude smaller than its radius (lateral extent) [131]. This justifies high-throughput microscopy as an examination method for mature biofilms, which provides images of the biofilm viewed from the top (or bottom). Similarly, it motivates the use of computationally inexpensive mathematical models posed in two-dimensional spatial domains in which the biofilm has no resolvable depth. However, colony biofilms can generate significant thickness, and form nutrient gradients along the z-direction, which significantly affects biofilm growth. For example, nitrogen diffuses upwards from the agar-solidified growth medium, oxygen diffuses downwards from the biofilm surface [82,83]. In single-strain colony biofilms, these gradients lead to division of labour among genotypically identical cells along this axis [83], thus raising questions about the validity of the commonly used ‘bird's eye perspective’ approach in which biofilms are essentially treated as a two-dimensional object viewed from above (or below). Future studies should address these questions by investigating how well ‘flat’ images capture the true three-dimensional structure of a biofilm across a wide range of contexts and employ mathematical models on three-dimensional spatial domains, albeit the latter remains a significant challenge due to the high computational cost of numerically solving such models.

Applications of the colony biofilm model are often limited to the number of different residents introduced to the inoculum. By contrast, biofilms in biological, industrial and medical settings can consist of a much larger number of residents [132,133]. Challenges exist both in ensuring suitable and ecologically relevant growth conditions for multiple members and with the examination of the spatial distribution of the residents in mature biofilms. High-throughput examination of biofilms using microscopy (often stereomicroscopy) relies on the expression of fluorescent proteins by cells, which imposes limits on the number of observable biofilm residents in experiments. Future approaches should thus develop novel methods that enable detailed examination of spatial patterns in biofilms comprising multiple residents. Mathematical modelling could play a crucial role in any extension to more diverse communities, because tracking large numbers of genetically different cell lineages *in silico* is not difficult [44].

Finally, biofilm formation in the laboratory is commonly investigated under conditions that approximate environmental homogeneity (homogeneous growth substrate, constant temperature, constant humidity, etc.). However, outside the laboratory, biofilms typically experience spatially and temporally heterogeneous environments, such as temporal fluctuations in temperature and spatial variation in nutrient availability. Limited knowledge exists regarding the impact of environmental heterogeneity on colony biofilms [44,87,134]. Thus a significant challenge for future work would be the translation of current knowledge to more realistic environments characterized by heterogeneity.

**Table 1.** Overview of reviewed papers. The main literature reviewed in this paper is classified according to topic, organism(s) and model(s) used. Only papers including mathematical modelling approaches are shown. PDE: partial differential equations; ODE: ordinary differential equations; SPDE: stochastic partial differential equations; IBM: individual-based models; CA: cellular automaton; DD: deterministic dynamics; RD: random dynamics; RICs: random initial conditions. Asterisks for IBMs and CAs indicate hybrid models in which IBMs and CAs are coupled with continuum equations to resolve dynamics of other quantities of interests (e.g. nutrients).

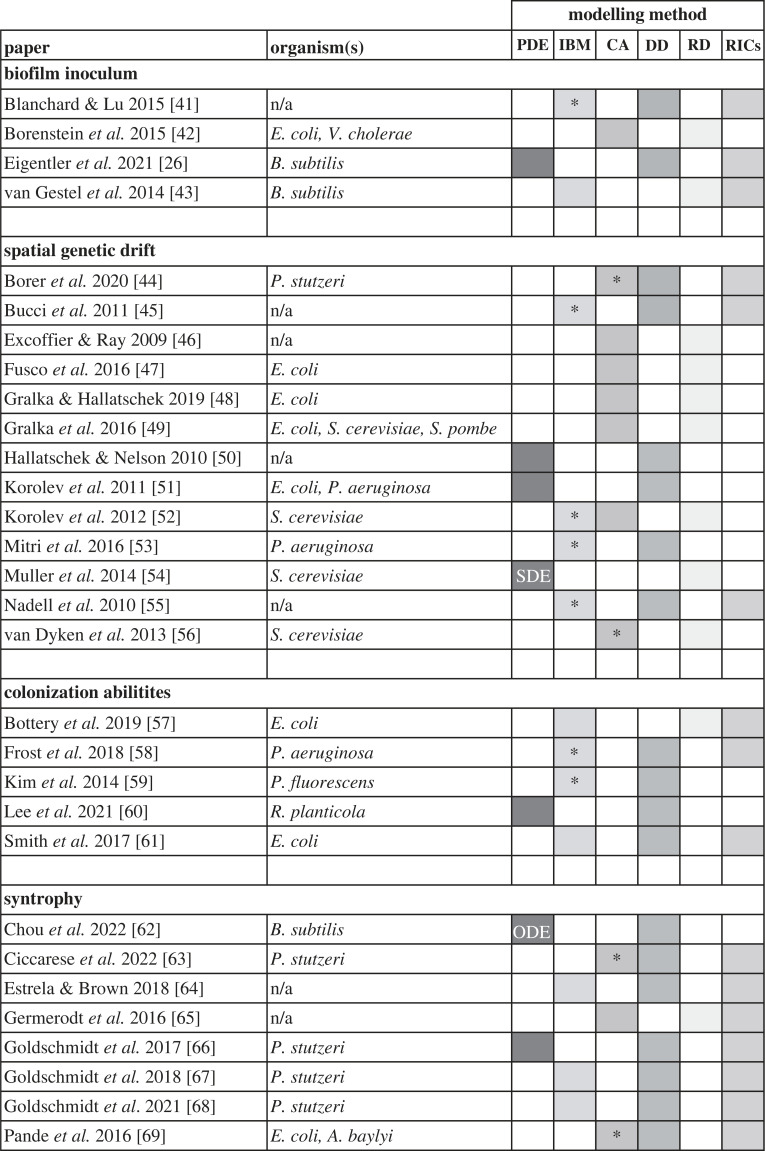

## Data Availability

This article has no additional data.
